# Food Group Intake and Micronutrient Adequacy in Adolescent Girls

**DOI:** 10.3390/nu4111692

**Published:** 2012-11-12

**Authors:** Lynn L. Moore, Martha R. Singer, M. Mustafa Qureshi, M. Loring Bradlee, Stephen R. Daniels

**Affiliations:** 1 Preventive Medicine & Epidemiology, Department of Medicine, Boston University School of Medicine, 72 East Concord St, Boston, MA 02118, USA; Email: msinger@bu.edu (M.R.S.); mustafaq@bu.edu (M.M.Q.); lbradlee@bu.edu (M.L.B.); 2 Department of Pediatrics, University of Colorado School of Medicine and The Children’s Hospital, 13123 E. 16th Avenue, Aurora, CO 80045, USA; Email: Stephen.Daniels@childrenscolorado.org

**Keywords:** nutrient adequacy, dietary intake, adolescents, longitudinal study

## Abstract

This study explores the contribution of food group intakes to micronutrient adequacy among 2379 girls in the National Growth and Health Study during three age periods (9–13, 14–18, and 19–20 years). Data on food and nutrient intakes from 3-day diet records over 10 years were used to estimate mean intakes and percent meeting *Dietary Guidelines *(*DGA*) recommendations for food intakes and Institute of Medicine’s recommendations for vitamins and minerals. More than 90% of girls failed to consume the recommended amounts of fruit, vegetables and dairy; 75% consumed less than the recommended amounts in the “meat” group. The vast majority of girls of all ages had inadequate intakes of calcium, magnesium, potassium, and vitamins D and E. In contrast, they consumed >750 kcal/day (~40% of total energy) from the DGA category of solid fat and added sugars, about five times the recommended maximum intakes. This study shows the importance of consuming a variety of foods in all five food groups, including those that are more energy dense such as dairy and meats, in order to meet a broad range of nutrient guidelines. Diet patterns that combined intakes across food groups led to greater improvements in overall nutritional adequacy.

## 1. Introduction

The Institute of Medicine sets forth recommended nutrient reference values known as Dietary Reference Intakes (DRIs) based on age, gender, and lifestage [1]. The United States Department of Agriculture’s (USDA) *Dietary Guidelines for Americans (DGA)* urge the consumption of nutrient-rich foods rather than the intake of supplements to meet these recommendations [2]. There has been growing concern, however, that the diets of most Americans fail to meet the recommended intakes of key nutrients [3]. Eating patterns are established early in life and this is of particular concern since food consumption patterns serve as the basis for nutrient adequacy. In one early study, only about one percent of American youth met the DGA in all food groups while 16% failed to meet the guidelines in any food group [4]. A later publication from the 2001–2004 *National Health and Nutrition Examination Surveys* confirmed that this trend continues [5].

The 1987–1988 USDA’s *Nationwide Food Consumption Survey* identified vitamins A and E, calcium, magnesium, and zinc as nutrients of concern for all adolescents, with females also having low intakes of phosphorus and iron [6]. Subsequent surveys have identified suboptimal intakes of folate and vitamin C as problematic [7,8,9]. In 2004 and 2005, the nationally representative *School Nutrition Dietary Assessment Study *found that 15% or more of school-aged children had inadequate intakes of vitamins A, C and E, magnesium, phosphorous, and zinc; levels of inadequacy increased with age, and among high school girls, there was also inadequate consumption of vitamin B6, folate, thiamin, calcium and iron [10]. Previously published data from ten years of follow up in the longitudinal *National Heart Lung and Blood Institute’s Growth and Health Study* (NGHS) showed that adolescent girls did not consume enough of the vitamins A, D, and E, as well as calcium and magnesium [11]. Most recently, the 2010 Dietary Guidelines Advisory Committee concluded that calcium, potassium, and vitamin D were nutrients of concern for both children and adults [12].

A few studies have examined the contribution of individual food groups to nutrient adequacy but the role of eating patterns has been rarely examined in either children or adults [13,14,15,16]. One such study of young adults demonstrated that those who met the DGA in all five major food groups were more likely to have adequate intakes of several important nutrients [17].

The goal of the current analysis is to explore the contribution of individual USDA Food Pyramid groups and subgroups as well as combined food group intakes to micronutrient adequacy in adolescent girls. The potential role of discretionary calories to overall nutritional adequacy will also be explored.

## 2. Experimental Section

### 2.1. Participants

The NGHS enrolled a total of 2379 black and white girls, ages 9–10 years at baseline, and followed them annually for 10 years. For the current analyses, we included girls during three different age periods: ages 9–13, 14–18 and 19–20 years. These ages were chosen to reflect ages at which the Dietary References Intakes change. A total of 2360, 2101 and 999 girls with diet record information in the three age groups, respectively, are included in these analyses. Dietary data from pregnant and lactating women were excluded from the analyses. Details of the study design and methods have been previously published [18,19]. These secondary analyses were reviewed and approved by the Boston University Institutional Review Board.

### 2.2. Measurements

#### 2.2.1. Dietary Assessment

Usual dietary intake was estimated using multiple sets of three-day diet records collected annually on two weekdays and one weekend day during study years 1–5, 7, 8, and 10. Data from each three day set were averaged and then all sets of diet records collected within each age period were further averaged. Of all diet records collected, more than 96% contained 3 days of data; less than 1% of the sets had only 1 day of dietary data. Data were analyzed for nutrient content using the Nutrition Data System (NDS) of the University of Minnesota [20]. The NDS nutrient database appropriate to the year of collection was used to calculate nutrient intakes for all foods consumed (excluding dietary supplements).

Food Pyramid servings were derived from the NDS output, based on disaggregated food and ingredient codes (including those from mixed dishes). These codes were linked by the authors with those of the USDA’s “*Pyramid Servings Database for USDA Survey Food Codes*, version 2” [21]. Exact matches between the two databases were found for approximately 70% of codes; for the remaining codes, a food with similar nutrient content was identified and the estimated Pyramid servings were then adjusted to reflect the differences in nutrient content. The final Food Pyramid data set included each subject’s intake in the five major food groups (*i.e.*, fruit, vegetables, dairy, meat/other proteins, grains) and all subgroups including intakes of solid fat and added sugars (SoFAS), as defined in the 2010 DGA [2]. Solid fats include shortenings and hard margarines as well as excess calories consumed by choosing higher-fat forms of dairy and meats (e.g., whole milk, chicken with skin) rather than available lower-fat forms. Added sugars include syrups and sugars that are added to foods in processing or preparation.

#### 2.2.2. Nutrient Intake Reference Values

As put forth in the DRI guidelines, the Estimated Average Requirement (EAR) is the average daily nutrient intake level that is estimated to meet the needs of 50% of healthy individuals. For potassium, an EAR has not yet been established and the adequate intake (AI) level is used instead. The AIs for potassium are based on a level of dietary intake that should maintain optimal blood pressure levels, lower the risk of recurrent kidney stones, and possibly reduce bone loss. Those consuming 100% of the age-specific EAR or AI were considered to meet the guidelines.

#### 2.2.3. Physical Activity

Physical activity was assessed in this study using a validated questionnaire [22] that was used to derive an estimate of metabolic equivalent (MET) times per day [23]. We then classified girls as sedentary who reported an average MET level of less than 3.0, moderately active for those with a MET level of 3.0–<6.0, or active for those with a MET level of ≥6.0 using CDC guidelines [24].

### 2.3. Statistical Analysis

To evaluate nutritional adequacy during three different age periods (9–13, 14–18, 19–20 years), mean intakes of vitamins A, B (thiamin, riboflavin, niacin, B6, B12), C, D and E, as well as calcium, magnesium, zinc, iron, phosphorus, and potassium, were estimated in addition to the percent of girls meeting the EAR (or the AI in the case of potassium). Secondly, the girls’ food consumption habits were assessed by comparing actual intake with that recommended by the DGA in various food groups [2]. Since foods classified as SoFAS in the Dietary Guidelines may contribute to an excess of calories without adding substantially to nutritional adequacy, the intake of SoFAS for girls during different age periods and having different levels of activity were compared with recommended intake amounts.

To evaluate the contribution of various food groups and subgroups (alone or consumed as a part of a pattern), each girl’s intake in USDA food groups and subgroups was classified as low, moderate, or high. For fruit, vegetables, meats and dairy, low, moderate and high intakes were defined as <50% of recommended (by the DGA), 50%–<75% of recommended and ≥75% of recommended, respectively. For grains, for which consumption levels were generally higher than those in other food groups, low, moderate and high intakes were defined as <75%, 75%–<100%, and ≥100% of recommended, respectively. Intake in several food subgroups were also classified as a percent of the recommended intake in the primary food group. For example, whole grain consumption was estimated as a percent of the recommended total grain intake for girls of a given age. Since whole grain consumption was very low, it was necessary to define low, moderate and high intakes using cutpoints of <5%, 5%–<10% and ≥10% of recommended total grain intake.

Nutritional adequacy associated with different levels of food group intake was measured as the extent to which these subjects met the EAR (or AI in the case of potassium) as set by the DRI for various nutrients. For each nutrient of interest, we determined the percent of girls who met the EAR associated with food intake during three age periods (9–13, 14–18, 19–20 years). The proportion of girls meeting the EAR levels for each nutrient was compared between food intake groups using a likelihood ratio test from the chi-squared analysis.

Each girl’s pattern of food intake was explored further by combining intakes in different food groups of varying nutrient and energy content (*i.e.*, nutrient-dense and energy-dense foods). There is no consensus definition for nutrient-dense or energy-dense foods [25]; rather, a food with greater nutrient density is one that provides a relatively higher nutrient content per serving than another food of a similar serving size. In general, all basic foods in the USDA food groups are considered nutrient dense foods, although they contain differing amounts of calories (e.g., an average serving of dairy has more calories than an average serving of vegetables). Thus, energy-dense foods are conceptualized as those supplying a relatively greater number of calories per serving than another food of a similar serving size. For each eating pattern, two types of nutrient-dense foods are included—one of which has greater energy density (*i.e.*, dairy, red meat, white meat) than the other (e.g., fruits, vegetables, whole grains). Each food group was dichotomized to reflect higher or lower intakes (exact cut-off points are specified in the legends for each figure) and then selected groups are cross-classified. For example, dairy and fruits and vegetables (FV) were divided into four mutually exclusive categories of intake: (a) Low Dairy/Low FV; (b) Low Dairy/High FV; (c) High Dairy/Low FV; and (d) High Dairy/High FV.

In the main analyses in Table 1, Table 2, Table 3, data are presented for all three age groups. At 14–18 years of age, recommended intake levels change for several nutrients and nutrient adequacy declines for some important nutrients (e.g., magnesium, zinc). For these reasons, and because 14–18 years of age serves as a representative portion of the adolescent period, subsequent analyses focus on girls during that age period. All analyses were carried out using SAS, version 9.2 (SAS Institute, Cary, NC, USA).

## 3. Results

In Table 1, the EARs for 14 nutrients of interest (and the AI for potassium) are shown for girls during three age periods. Mean intakes for some nutrients were well above EAR levels while those for vitamin D, E, calcium, magnesium, potassium, and phosphorus (for all except the oldest girls) were far below established guidelines. In general, the percent of girls failing to meet the EAR increased with age. Adequacy of calcium improved with age since the EAR dropped from 1100 mg in the younger age groups to 800 mg at age 19 and older. By mid-adolescence, many girls failed to meet EAR recommendations; more than 90% failed to meet the guidelines for vitamins D and E, magnesium, and potassium. For many nutrients, results were similar for black and white girls. Blacks, however, had lower intakes of many dairy-related vitamins and minerals. Most notably, only 5.2% of black girls in mid-adolescence (*vs.* 17.9% of whites) met the EAR for calcium.

**Table 1:** Mean micronutrient intake and percent of girls meeting estimated average requirements (EAR), stratifying by age and race.

		Vit A (RAE)	Thiamin (mg)	Riboflavin (mg)	Niacin (mg)	Vit B6 (mg)	Vit B12 (μg)	Vit C (mg)	Vit D (μg)
**Girls**		**Established Estimated Average Requirements (EAR)**							
9–13 years		420	0.7	0.8	9	0.8	1.5	39	10.0
14–18 years		485	0.9	0.9	11	1.0	2.0	56	10.0
19–20 years		500	0.9	0.9	11	1.1	2.0	60	10.0
		**Intakes for NGHS Girls, by Age**							
**All**	***n***	**( *geometric mean ± s.d.* )**							
9–13 years	2360	579 ± 1.6	1.5 ± 1.3	1.7 ± 1.3	17.7 ± 1.3	1.4 ± 1.3	3.9 ± 1.5	84.9 ± 1.6	4.7 ± 1.6
14–18 years	2101	494 ± 1.8	1.5 ± 1.4	1.6 ± 1.4	17.5 ± 1.4	1.3 ± 1.4	3.2 ± 1.7	85.1 ± 1.9	3.5 ± 1.9
19–20 years	999	441 ± 2.1	1.5 ± 1.5	1.5 ± 1.5	18.3 ± 1.5	1.4 ± 1.6	2.9 ± 1.9	72.4 ± 2.3	3.1 ± 2.4
**All**		**% of Girls Meeting EAR, by Age and Race**							
9–13 years	2360	77.8%	99.9%	99.7%	99.4%	97.0%	99.3%	94.5%	1.9%
14–18 years	2101	53.2%	93.4%	94.1%	93.9%	81.3%	84.4%	76.8%	2.4%
19–20 years	999	46.3%	88.1%	88.0%	90.9%	69.5%	76.0%	60.0%	5.1%
*p-value*		*<0.0001*	*0.0469*	*0.0468*	*0.2036*	*<0.0001*	*<0.0001*	*<0.0001*	*<0.0001*
**White**									
9–13 years	1157	85.2%	100.0%	99.9%	99.3%	96.5%	99.1%	92.9%	2.9%
14–18 years	1014	63.4%	93.4%	95.4%	91.7%	78.3%	82.1%	71.9%	3.9%
19–20 years	446	53.1%	89.2%	91.0%	90.4%	65.5%	75.8%	52.2%	7.6%
*p-value*		*<0.0001*	*0.2995*	*0.4448*	*0.3698*	*<0.0001*	*0.0015*	*<0.0001*	*0.0008*
**Black**									
9–13 years	1203	70.7%	99.8%	99.5%	99.5%	97.3%	99.4%	96.1%	0.9%
14–18 years	1087	43.7%	93.4%	92.8%	95.9%	84.0%	86.7%	81.4%	1.1%
19–20 years	553	40.7%	87.2%	85.5%	91.3%	72.7%	76.1%	66.2%	3.1%
*p-value*		*<0.0001*	*0.1529*	*0.0969*	*0.4501*	*0.0005*	*0.0014*	*<0.0001*	*0.0030*
		**Vit E (mg)**	**Ca (mg)**	**Mg (mg)**	**Zn (mg)**	**Fe (mg)**	**P (mg)**	**K (mg) ^1^**
**Girls**		**Established Estimated Average Requirements (EAR)**						
9–13 years		9.0	1100	200	7.0	5.7	1055	4500
14–18 years		12.0	1100	300	7.3	7.9	1055	4700
19–20 years		12.0	800	255	6.8	8.1	580	4700
		**Intakes for NGHS Girls, by Age**						
**All **	***n***	**( *geometric mean ± s.d.* )**						
9–13 years	2360	5.4 ± 1.5	756 ± 1.4	208 ± 1.3	9.2 ± 1.3	11.7 ± 1.3	1056 ± 1.3	1970 ± 1.3
14–18 years	2101	5.4 ± 1.5	672 ± 1.5	198 ± 1.4	8.6 ± 1.4	11.5 ± 1.4	995 ± 1.4	1883 ± 1.4
19–20 years	999	4.9 ± 1.6	632 ± 1.6	196 ± 1.5	8.5 ± 1.5	11.7 ± 1.5	991 ± 1.4	1863 ± 1.5
**All**		**% of Girls Meeting EAR, by Age and Race**						
9–13 years	2360	8.5%	11.3%	56.5%	85.5%	99.6%	50.8%	0.0%
14–18 years	2101	2.4%	11.3%	9.0%	69.9%	88.7%	42.8%	0.1%
19–20 years	999	1.8%	33.2%	25.1%	73.6%	81.7%	92.1%	0.9%
*p-value*		*<0.0001*	*<0.0001*	*<0.0001*	*0.0002*	*0.0007*	*<0.0001*	*<0.0001*
**White**								
9–13 years	1157	4.2%	17.6%	60.4%	84.2%	99.6%	56.4%	0.0%
14–18 years	1014	1.5%	17.9%	11.7%	65.4%	89.6%	48.6%	0.1%
19–20 years	446	0.9%	44.4%	31.4%	70.4%	84.1%	93.7%	1.4%
*p-value*		*0.0072*	*<0.0001*	*<0.0001*	*0.0020*	*0.0748*	*<0.0001*	*<0.0001*
**Black **								
9–13 years	1203	12.6%	5.3%	52.8%	86.8%	99.7%	45.4%	0.0%
14–18 years	1087	3.2%	5.2%	6.4%	74.2%	88.0%	37.4%	0.1%
19–20 years	553	2.5%	24.2%	20.1%	76.3%	79.8%	90.8%	0.5%
*p-value*		*<0.0001*	*<0.0001*	*<0.0001*	*0.0665*	*0.0089*	*<0.0001*	*0.0382*

^1^ No EAR available for Potassium so these data reflect Adequate Intake (AI) levels.

Table 2 shows the recommended servings per day in the five major USDA food groups. The observed mean intakes at each age were far below recommended consumption levels for all except grains. Less than 10% of all girls consumed the recommended amounts of fruit, vegetables or dairy and only about 25% overall had adequate intakes in the meat group. Meat group consumption was much lower among whites compared with blacks while the opposite was true for dairy.

**Table 2:** USDA recommended food groups intake compared to NGHS girls, stratifying by age and race.

Subjects		Fruit	Vegetables	Dairy	Meats	Grains
**All Girls**		**Recommended food servings ^1,2^**				
9–13 years		3.0	4.0	3.0	5.0	5.0
14–18 years		3.0	5.0	3.0	5.0	6.0
19–20 years		4.0	5.0	3.0	5.5	6.0
		**Reported intakes at each age**				
**All**	***n***	**(*geometric mean ± s.d.*)**				
9–13 years	2360	1.1 ± 1.4	1.9 ± 1.3	1.6 ± 1.3	3.9 ± 1.4	6.1 ± 1.3
14–18 years	2101	1.0 ± 1.6	2.2 ± 1.4	1.5 ± 1.4	3.6 ± 1.5	5.9 ± 1.4
19–20 years	999	0.9 ± 1.7	2.3 ± 1.6	1.3 ± 1.5	3.6 ± 1.7	5.9 ± 1.6
		**% NGHS girls meeting recommended food intakes, by age and race**				
**All**						
9–13 years	2360	4.6%	2.8%	6.4%	24.8%	77.2%
14–18 years	2101	7.8%	2.9%	7.7%	24.3%	51.6%
19–20 years	999	5.6%	7.8%	8.9%	24.3%	51.3%
*p-trend*		*<0.0001*	*<0.0001*	*0.0445*	*0.9342*	*<0.0001*
**White**						
9–13 years	1157	5.6%	1.8%	11.7%	11.5%	79.3%
14–18 years	1014	10.8%	1.5%	13.3%	9.4%	57.9%
19–20 years	446	7.2%	6.1%	16.1%	10.3%	57.9%
*p-trend*		*0.0001*	*<0.0001*	*0.0817*	*0.3094*	*<0.0001*
**Black**						
9–13 years	1203	3.6%	3.8%	1.4%	37.6%	75.2%
14–18 years	1087	5.0%	4.2%	2.4%	38.2%	45.8%
19–20 years	553	4.3%	9.2%	3.1%	35.6%	45.9%
*p-trend*		*0.2703*	*<0.0001*	*0.0604*	*0.7221*	*<0.0001*

^1^ Sample servings: 1 fruit serving = 1 medium fruit (e.g., apple), 1/2 cup chopped, cooked, or canned fruit or 3/4 c. juice; 1 vegetable serving = 1 c. raw leafy vegetable, 1/2 c. raw/cooked other vegetables or 3/4 c. vegetable juice; 1 dairy serving = 1 cup milk/yogurt, 1.5 oz. natural/2.0 oz. processed cheese; 1 meat serving = 1 oz. lean meat, fish or poultry, 1/2 cup dry cooked beans, 1 egg, or 2 Tbs. peanut butter; 1 grain serving (whole and refined) = 1 slice bread, 1 cup dry cereal, 1/2 cup cooked rice, pasta, or cereal; ^2^ Based on recommended energy intake as determined by the USDA’s *Dietary Guidelines for Americans*, 2010 [2].

Table 3 first provides information from the DGA on the recommended intakes of total calories and discretionary calories from SoFAS [2] for sedentary, moderately active, and active girls in the three age groups. Secondly, the table shows data on estimated mean total daily energy intake during three age periods for NGHS girls as well as the number of calories and percent of total energy that were derived from SoFAS. For sedentary girls of these ages, the USDA recommends a maximum percent of intake from SoFAS ranging from 7.6% to 12.9% of total calories. For moderately active and active girls, the SoFAS allowance ranges from 8.9% to 13.8% of calories. In practice, these girls received about 40% of their total daily energy from SoFAS. At 9–13 years of age, for example, they consumed 42.0% (792 kcal/day) of their total daily energy (1877 kcal/day) from SoFAS, compared with the allowable 121 kcal/day for sedentary girls or 161 kcal/day for moderately active girls. Thus, these girls were consuming about five times the recommended amount of calories from solid fat and added sugars.

**Table 3:** USDA recommended energy and SoFAS intakes compared to NGHS girls, stratifying by activity level.

	USDA Recommended Intakes ^1^				
			Total Energy Intake	Allowance from SoFAS	
			(kcal/day)	(kcal/day)	(% of energy)
**Sedentary Girls**					
9–13 years			1600	121	7.6%
14–18 years			1800	161	8.9%
19–20 years			2000	258	12.9%
**Moderately Active Girls**					
9–13 years			1800	161	8.9%
14–18 years			2000	258	12.9%
19–20 years			2200	266	12.1%
**Active Girls**					
9–13 years			2000	258	12.9%
10–14 years			2400	330	13.8%
19–20 years			2400	330	13.8%
		**NGHS Girls Intakes**			
			**Total Energy Intake**	**Intake from SoFAS**	
	***n***		**(mean ± s.d.)**	**(mean ± s.d.)**	**(% of energy)**
**All**					
9–13 years	2360		1877 ± 399	792 ± 218	42.0 ± 5.4
14–18 years	2101		1875 ± 487	759 ± 259	40.1 ± 7.2
19–20 years	999		1920 ± 582	767 ± 310	39.5 ± 9.4
**Sedentary**					
9–13 years	892		1864 ± 381	790 ± 206	42.2 ± 5.4
14–18 years	1848		1874 ± 491	775 ± 257	41.0 ± 6.9
19–20 years	776		1938 ± 589	801 ± 310	41.0 ± 8.8
**Moderately Active**					
9–13 years	1187		1873 ± 402	790 ± 221	41.9 ± 5.5
14–18 years	331		1858 ± 452	715 ± 257	37.9 ± 7.6
19–20 years	139		1864 ± 535	723 ± 296	38.3 ± 10.1
**Active**					
9–13 years	271		1927 ± 442	803 ± 235	41.3 ± 5.4
14–18 years	46		2009 ± 518	718 ± 297	35.0 ± 8.3
19–20 years	96		1849 ± 584	671 ± 352	35.0 ± 11.1

^1 ^Based on recommended energy intake as determined by the USDA’s *Dietary Guidelines for Americans*, 2010 [2].

Table 4 shows the contribution of the five major food groups to adequacy of intake for eight key nutrients. While vitamin A is widespread in the food supply in the form of either pre-formed vitamin A or provitamin A carotenoids, the largest individual food group contributor to adequacy is dairy; more than 90% of girls in the higher dairy intake group met the EAR for vitamin A. For vitamin B6, more than 90% of girls with high intakes in any of the major food groups met the EAR (compared with about 60%–75% of girls with low intakes). Dairy, meats, and grains supported vitamin B12 adequacy while fruits and vegetables (FVs) contributed more vitamin C. Inadequate magnesium intake was widespread in these girls but this was particularly true for those with low intakes of fruit, vegetables and dairy. Girls with low meat intakes were most likely to have problems with adequate zinc consumption. Finally, we found that overall adequacy of calcium consumption was very low; none of the girls with low dairy intake (<50% of recommended) met the 2010 EAR of 1100 mg/day. 

**Table 4:** Percent of 14–18-year-old girls meeting micronutrient EARs by level of food group intake.

Food Intake ^1^	*n*	Vit A (RAE)	Vit B6 (mg)	Vit B12 (μg)	Vit C (mg)	Ca (mg)	Mg (mg)	Zn (mg)	P (mg)
		**% Meeting EAR**							
**Fruit**									
Low	1436	47.5%	77.2%	84.3%	67.2%	8.6%	5.1%	67.6%	38.9%
Moderate	326	60.1%	87.7%	84.4%	95.4%	13.5%	7.7%	75.8%	47.6%
High	339	70.8%	92.3%	85.0%	99.7%	20.4%	26.8%	74.0%	54.6%
*p-value*		*<0.0001*	*0.0001*	*0.9589*	*<0.0001*	*<0.0001*	*<0.0001*	*0.0026*	*<0.0001*
**Vegetables**									
Low	1330	49.7%	72.7%	81.7%	71.8%	11.4%	5.7%	62.8%	35.3%
Moderate	549	55.7%	94.7%	88.0%	84.0%	10.2%	8.4%	78.9%	49.5%
High	222	68.0%	99.1%	91.9%	89.2%	13.5%	30.2%	90.5%	70.7%
*p-value*		*<0.0001*	*<0.0001*	*<0.0001*	*<0.0001*	*0.4162*	*<0.0001*	*<0.0001*	*<0.0001*
**Dairy**									
Low	1105	32.1%	73.3%	73.4%	74.6%	0.0%	3.4%	56.3%	15.9%
Moderate	561	66.1%	86.3%	94.7%	77.2%	1.4%	6.1%	78.4%	54.9%
High	435	90.1%	94.9%	99.3%	82.1%	52.6%	26.9%	93.6%	95.4%
*p-value*		*<0.0001*	*<0.0001*	*<0.0001*	*0.0059*	*<0.0001*	*<0.0001*	*<0.0001*	*<0.0001*
**Meat Group**									
Low	477	54.5%	60.4%	63.1%	67.7%	12.6%	7.1%	37.3%	28.3%
Moderate	620	52.4%	77.9%	83.6%	75.3%	11.1%	6.0%	61.3%	32.9%
High	1004	53.1%	93.2%	95.1%	82.1%	10.8%	11.8%	90.7%	55.8%
*p-value*		*0.7850*	*<0.0001*	*<0.0001*	*<0.0001*	*<0.0001*	*0.0001*	*<0.0001*	*<0.0001*
**Grains**									
Low	421	30.9%	62.7%	75.3%	66.0%	3.1%	1.9%	44.4%	11.2%
Moderate	595	46.6%	77.1%	80.8%	73.5%	4.5%	3.4%	62.4%	28.4%
High	1085	65.5%	90.7%	90.0%	82.9%	18.2%	14.8%	84.0%	63.0%
*p-value*		*<0.0001*	*<0.0001*	*<0.0001*	*<0.0001*	*<0.0001*	*<0.0001*	*<0.0001*	*<0.0001*

^1^ For grains, low, moderate, and high intakes: <75%, 75%–<100%, ≥100% of DGA recommended amounts, respectively. For all other food groups, low, moderate, and high intakes: <50%, 50%–<75%, and ≥75% of recommended amounts.

The foods consumed within individual food groups make different contributions to micronutrient adequacy. Therefore in Supplemental Tables S1–S5, we provide additional data on the effects of various USDA food subgroups on 12 nutrients of interest for girls during mid-adolescence. These tables show that dietary variety within the major food groups is perhaps nearly as important as variety across food groups. For example, red meat and fish contain more vitamin B12 than does chicken, and different FV subgroups contribute very differently to adequacy of vitamin A intake. 

**Figure 1:**
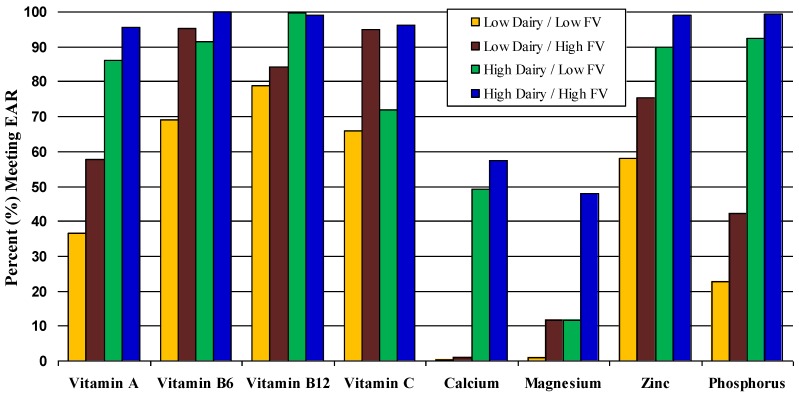
Percent of 14–18 year-old girls meeting EAR according to combined effects of fruits and vegetables and whole dairy. Low (*vs.* high) fruit and vegetable intake: <50% (*vs.* ≥50%) of recommended 8 servings of total fruit and vegetables/day. Low (*vs.* high) dairy: <75% (*vs.* ≥75%) of recommended 3 dairy servings/day.

**Figure 2:**
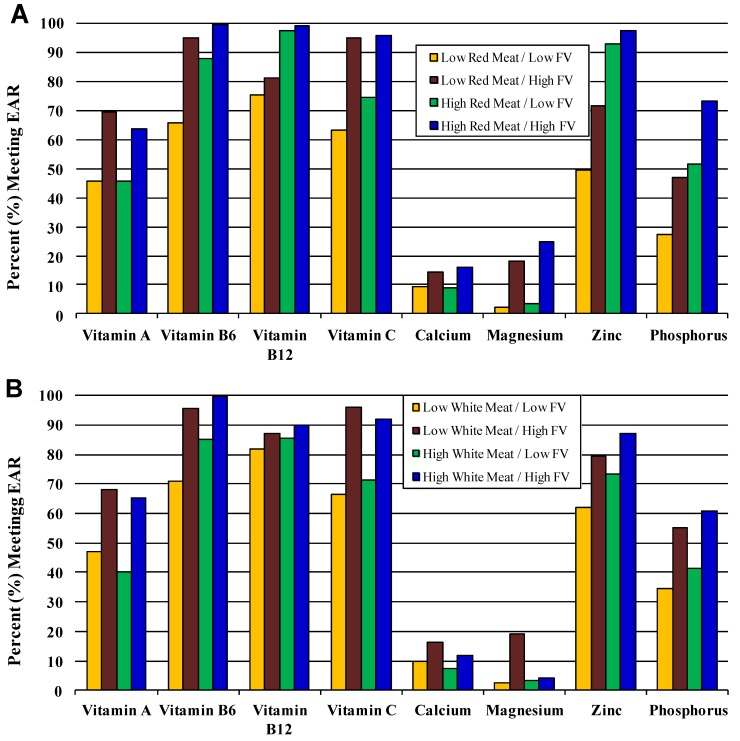
Percent of 14–18 year-old girls meeting EAR according to combined effects of fruits and vegetables and (**A**) red meat and (**B**) white meat (poultry and fish). Low (*vs.* high) fruit and vegetable intake ≤50% (*vs.* ≥50%) of recommended 8 servings of total fruit and vegetables/day. Low (*vs.* high) dairy: <75% (*vs.* ≥75%) of recommended 3 dairy servings/day. Low (*vs.* high) red meat: <75% (*vs.* ≥75%) of recommended 5 one-ounce servings of total meat/day. Low (*vs.* high) white meat (poultry and fish): <40% (*vs.* ≥40%) of 5 one-ounce servings of total meat/day.

Finally, we explored the effects of selected eating patterns on micronutrient adequacy in Figure 1 and Figure 2. Too few girls met the EAR for vitamin E or the AI for potassium to display graphically. Figure 1 explores the combined effects dairy products and FVs on these nutrients in all girls ages 14–18 years. In this figure each girl is classified into one of four patterns: (a) high intakes of both dairy and FVs, (b) high intakes of dairy but low intakes of FVs, (c) low intakes of dairy with high intakes of FVs, and (d) low intakes of both. Cutpoints for these categories are given in the footnotes. It is apparent from this figure that girls with higher intakes of dairy and FVs are much more likely to meet the EAR for a wide range of nutrients. In contrast, nearly all of the girls who consumed less than 75% of the recommended servings of dairy per day failed to meet the new EAR for calcium.

Figure 2 (A and B) compare two eating patterns, one including red meat and the other with poultry and fish (white meat). Each of these meat subgroups is shown in combination with FV intakes. Overall, these combined patterns were associated with a higher proportion of all girls aged 14 to 18 years meeting the EAR for B-vitamins, vitamin C (from the FV content) and zinc. However, higher red meat consumption benefitted vitamin B12, zinc and phosphorus intakes more than did white meat. Similarly, red meat when combined with higher intakes of FVs also led to a greater number of girls meeting the EAR for magnesium, zinc, phosphorus, and B-vitamins. Additional dietary patterns are explored in Supplemental Figures S1–S5.

## 4. Discussion

This study confirms that micronutrient inadequacy increases as girls moved from early to mid-adolescence and beyond. By 14–18 years of age, almost 90% failed to meet the EAR for the intake of calcium and more than 90% failed to meet guidelines for magnesium, potassium and vitamins D and E. These estimates are similar to those from the 2005 and 2006 NHANES data [9]. Large numbers of girls also had inadequate intakes of vitamin A, zinc and phosphorus and even vitamins B6, B12 and C.

Nutrient density is a relative concept. Foods in the same food group may differ in nutrient density as well as in energy density. Grains, for instance, can be consumed in both energy rich and less energy-dense forms. This study demonstrates the potentially important contribution of nutrient-dense eating patterns to micronutrient adequacy amongst adolescent girls. It also points to the role of excess consumption of discretionary calories in the form of solid fat and added sugars to micronutrient inadequacy. While the 2010 DGA cites that SoFAS account for approximately 35% of calories consumed by Americans [2], girls in this prospective study consumed approximately 40% of their total daily calories from these energy dense, nutrient-poor sources. The current finding is consistent with published cross-sectional data from younger school-age subjects in NHANES 1999–2004 [26]. The excess consumption of such discretionary calories in place of more nutrient-dense foods likely plays an important role in the failure of children and adolescents to meet DGA and IOM dietary recommendations for both foods and nutrients. 

The contribution of food groups and subgroups to micronutrient adequacy depends on several factors—nutrient composition of the individual foods in a group/subgroup, the mix of foods eaten within the food group/subgroup, the amount of each consumed, and the correlation of intakes within and between food groups. For example, whole grain consumption might be correlated with fruit consumption among individuals focusing on a healthy diet while higher red meat intake might be correlated with consumption of starchy vegetables (e.g., French fries) in other individuals. Thus, correlated eating patterns may obscure the true nutrient contribution of individual foods or food groups. To address this issue, we constructed simple eating patterns that reflected the different ways in which foods may be eaten (e.g., some girls with high red meat intakes consume few FVs while others may consume more). In this way, we were able to see the effects of red meat independent of its associations with FV intakes. 

Dairy is not only a rich source of many nutrients [27] but its association with consumption of whole and fortified grains (*i.e.*, through breakfast cereals) may explain some of its broad-ranging micronutrient benefits. In this study, all girls with low dairy intakes failed to meet the EAR for calcium. This is of particular concern during adolescence when peak bone mass accrual occurs [28,29,30]. Low dairy intake is also associated with vitamin D deficiency and with the consumption of sugar-sweetened and/or carbonated beverages [31] which also may adversely affect bone accrual by the pairing low calcium consumption with low vitamin D and high phosphorus intakes [32,33,34,35]. 

Red meat consumption had beneficial effects on adequacy of intake of vitamins B6 and B12 as well as phosphorus and zinc. This is consistent with recent analyses from NHANES in which the consumption of red meat amongst children and adolescents was associated with greater nutrient adequacy for a number of micronutrients [36]. Among midadolescent girls with low red meat intakes, more than 30% failed to take in adequate amounts of these important B-vitamins. 

While the adolescent girls in this study had high intakes of grains, most of these were derived from refined sources. B-vitamins that are lost in the refinement of grains have been replaced through fortification since the 1940s. Whole grains have higher fiber and protein contents than refined grains and are also more important sources of magnesium and other nutrients and phytochemicals such as lignans. Thus, increasing the proportion of total grain intake from whole grain sources is an important target for dietary change during childhood and adolescence. 

The role of multivitamin use in micronutrient adequacy is unclear. In the NGHS, 36% of girls (42.7% of whites and 29.1% of blacks) at the end of the follow-up reported taking multivitamins. This is similar to recent data from the 1999–2004 NHANES, showing that 34.2% of children and adolescents took a multivitamin/mineral supplement (although only half of those did so daily) [37]. Multivitamin consumers tended to have better nutrition and healthier lifestyles overall suggesting that those who might benefit most from supplementation were least likely to take vitamins. In addition, whether multivitamin/mineral supplements provide health benefits that are equal to those of nutrient-rich foods is an open question. 

This study has a number of important strengths. It is a relatively large prospective study with dietary data from multiple sets of three-day diet records, yielding more precise estimates of dietary intake. Another important strength is the inclusion of USDA-defined Food Pyramid servings that were derived by the authors by linking NDS food codes with USDA Survey Food codes. These methods for deriving food groups/subgroups included disaggregating all mixed dishes into their component parts for the estimation of food group servings. While dietary intake was assessed using a gold-standard method for large-scale epidemiologic studies, there are still limitations associated with the use of self-reported diet records, particularly at younger ages. At the outset of the study, the girls were 9–10 years of age, an age at which accurate quantification of amounts consumed is difficult. While the children were encouraged to get details of recipes and food preparation from a parent, there is no guarantee that this was consistently done. The absence of such details is a likely source of nondifferential error in the estimation of food and nutrient intakes. All self-reported dietary assessment methods are also prone to bias. Overweight/obese girls may underreport the consumption of foods and nutrients that they believe to be associated with body fat. However, it has been shown that such bias is most correlated with the underreporting of snacks, rather than foods at meals [38]. Such underreporting could lead to an underascertainment of foods classified as SoFAS. Finally, although the study is larger than most prospective studies, particularly those with detailed diet records, its size is still somewhat limited for examining certain subgroups. For example, since whole grain consumption was quite low, it is not possible to demonstrate the contribution of intake levels that more closely match the Dietary Guideline recommendations.

## 5. Conclusions

This study illustrates that most adolescent girls fail to meet dietary intake recommendations in all major food groups except grains, with the vast majority of grains consumed being from refined sources (see Supplemental Tables). In addition, many of these girls failed to have adequate intakes of important micronutrients. This is most likely due to the overconsumption of nutrient-poor foods and excessive intakes of calories from solid fat and added sugars. This study suggests that energy-dense, nutrient-rich foods such as meats and dairy products, as well as less energy-dense, nutrient-rich foods such as fruits and vegetables make important contributions to micronutrient adequacy during critical adolescent periods. 

## Implications

Dietary guidance has far too often focused on restricting intakes of certain foods in an attempt to reduce the intake of dietary fats in particular. This study suggests that more appropriate guidance might be needed to encourage the consumption of a wide variety of nutrient-rich foods and to choose less energy-dense forms of nutrient-rich foods from all food groups.

## Supplementary Files

Supplementary File 1: Supplementary File (PDF, 294 KB)
