# Supplementary material for: Food Group Intake and Micronutrient Adequacy in Adolescent Girls
Source: Nutrients. 2012 Nov 12;4(11):1692–708. doi: 10.3390/nu4111692 (PMC3509514; doi:10.3390/nu4111692)

Supplementary Materials

Supplemental Table S1. Percent of 14–18 year-old girls meeting Estimated Average Requirements (EAR) for micronutrients according to fruit subgroup intake.

	Citrus, melon, berries			
	**Low [1]**	**Moderate [1]**	**High [1]**	***p-value***
	n = 794	*n = 924*	*n = 383*	
**Vit A (RAE)**	46.6%	54.7%	63.5%	*<0.0001*
**Vit B6 (mg)**	74.1%	84.0%	89.6%	*<0.0001*
**Vit B12 (µg)**	85.4%	84.2%	83.0%	*0.5579*
**Vit C (mg)**	48.1%	91.9%	100.0%	*<0.0001*
**Vit D (µg)**	2.4%	2.4%	2.6%	*0.9671*
**Vit E (mg)**	2.1%	2.1%	3.7%	*0.1927*
**Ca (mg)**	9.5%	10.5%	17.0%	*0.0004*
**Mg (mg)**	4.9%	8.4%	18.8%	*<0.0001*
**Zn (mg)**	67.8%	71.1%	71.5%	*0.2395*
**Iron (mg)**	84.1%	90.5%	94.0%	*<0.0001*
**P (mg)**	38.4%	43.7%	49.6%	*0.0010*
**K (mg) [2]**	0.0%	0.2%	0.0%	*0.2794*
	Other fruit			
	**Low [3]**	**Moderate [3]**	**High [3]**	***p-value***
	n = 958	*n = 839*	*n = 304*	
**Vit A (RAE)**	44.1%	57.1%	71.4%	*<0.0001*
**Vit B6 (mg)**	75.0%	84.9%	91.1%	*<0.0001*
**Vit B12 (µg)**	84.5%	83.7%	86.5%	*0.5036*
**Vit C (mg)**	65.9%	83.2%	93.8%	*<0.0001*
**Vit D (µg)**	2.4%	1.6%	4.9%	*0.0045*
**Vit E (mg)**	2.4%	2.2%	3.0%	*0.7256*
**Ca (mg)**	8.0%	11.3%	21.4%	*<0.0001*
**Mg (mg)**	5.0%	8.6%	22.7%	*<0.0001*
**Zn (mg)**	67.9%	69.0%	79.0%	*0.0009*
**Iron (mg)**	82.8%	92.5%	97.0%	*<0.0001*
**P (mg)**	38.1%	43.9%	54.6%	*<0.0001*
**K (mg) [2]**	0.1%	0.0%	0.3%	*0.2788*

[1] Low, moderate, high intakes: <10%, 10%–<40%, ≥40% of the recommended 3 servings of total fruit/day derived from citrus, melon, berries; [2] Percent meeting AI for potassium (K); [3] Low, moderate, high intakes: <10%, 10%–<40%, ≥40% of the recommended 3 servings of total fruit/day derived from other fruit.

Supplemental Table S2. Percent of 14–18 year-old girls meeting Estimated Average Requirements (EAR) for micronutrients according to vegetable subgroup intake.

	Tomatoes, dark green, deep yellow and orange vegetables			
	**Low** [1]	**Moderate** [1]	**High** [1]	***p-value***
	n = 1085	*n = 730*	*n = 286*	
**Vit A (RAE)**	41.4%	60.1%	80.4%	*<0.0001*
**Vit B6 (mg)**	74.4%	87.5%	91.3%	*<0.0001*
**Vit B12 (μg)**	80.4%	90.0%	85.7%	*<0.0001*
**Vit C (mg)**	68.2%	84.1%	90.9%	*<0.0001*
**Vit D (μg)**	2.7%	1.8%	3.2%	*0.3346*
**Vit E (mg)**	1.8%	2.2%	5.2%	*0.0024*
**Ca (mg)**	8.5%	12.9%	17.8%	*<0.0001*
**Mg (mg)**	5.3%	9.3%	22.4%	*<0.0001*
**Zn (mg)**	60.3%	80.3%	80.1%	*<0.0001*
**Iron (mg)**	82.2%	95.6%	95.8%	*<0.0001*
**P (mg)**	32.1%	51.0%	62.6%	*<0.0001*
**K (mg)** [2]	0.2%	0.0%	0.0%	*0.3917*
	Other non-starchy vegetables			
	**Low** [3]	**Moderate** [3]	**High** [3]	***p-value***
	n = 1058	*n = 755*	*n = 288*	
**Vit A (RAE)**	46.1%	58.0%	66.7%	*<0.0001*
**Vit B6 (mg)**	75.2%	85.3%	92.7%	*<0.0001*
**Vit B12 (μg)**	82.9%	86.1%	85.8%	*0.1436*
**Vit C (mg)**	71.3%	81.1%	86.1%	*<0.0001*
**Vit D (μg)**	2.7%	2.1%	2.4%	*0.7721*
**Vit E (mg)**	1.6%	2.4%	5.2%	*0.0018*
**Ca (mg)**	11.3%	10.3%	13.5%	*0.3404*
**Mg (mg)**	6.3%	8.2%	20.8%	*<0.0001*
**Zn (mg)**	63.6%	75.2%	79.2%	*<0.0001*
**Iron (mg)**	84.0%	91.9%	97.6%	*<0.0001*
**P (mg)**	36.4%	46.1%	57.6%	*<0.0001*
**K (mg)** [2]	0.1%	0.0%	0.4%	*0.2667*
	Potatoes & other starchy vegetables			
	**Low** [4]	**Moderate** [4]	**High** [4]	***p-value***
	n = 472	*n = 1043*	*n = 586*	
**Vit A (RAE)**	55.5%	53.9%	50.2%	*0.1859*
**Vit B6 (mg)**	64.8%	81.1%	94.7%	*<0.0001*
**Vit B12 (ug)**	78.6%	84.4%	89.3%	*<0.0001*
**Vit C (mg)**	71.4%	76.4%	81.9%	*0.0003*
**Vit D (μg)**	3.4%	2.2%	2.1%	*0.2982*
**Vit E (mg)**	1.5%	1.3%	5.1%	*<0.0001*
**Ca (mg)**	14.6%	11.0%	9.0%	*0.0162*
**Mg (mg)**	8.9%	6.8%	13.0%	*0.0002*
**Zn (mg)**	56.6%	69.7%	81.1%	*<0.0001*
**Iron (mg)**	82.8%	89.2%	92.7%	*<0.0001*
**P (mg)**	33.5%	41.2%	53.1%	*<0.0001*
**K (mg)** [2]	0.2%	0.0%	0.2%	*0.3642*

[1] Low, moderate, high: <10%, 10%–<20%, ≥20% of the recommended 5 servings of total vegetable/day derived from tomatoes, dark green, deep yellow and orange vegetables; [2] Percent meeting AI for potassium (K); [3] Low, moderate, high: <10%, 10%–<20%, ≥20% of the recommended 5 servings of total vegetable/day derived from other non-starchy vegetables; [4] Low, moderate, high: <10%, 10%–<30%, ≥30% of the recommended 5 servings of total vegetable/day derived from potatoes & other starchy vegetables subgroup.

Supplemental Table S3. Percent of 14–18 year-old girls meeting Estimated Average Requirements (EAR) for micronutrients according to dairy subgroup intake.

	Milk subgroup			
	**Low** [1]	**Moderate** [1]	**High** [1]	***p-value***
	n = 830	*n = 862*	*n = 409*	
**Vit A (RAE)**	23.3%	64.3%	90.7%	*<0.0001*
**Vit B6 (mg)**	70.5%	85.6%	93.9%	*<0.0001*
**Vit B12 (µg)**	70.5%	91.0%	99.0%	*<0.0001*
**Vit C (mg)**	74.5%	78.4%	78.2%	*0.1161*
**Vit D (µg)**	0.1%	0.0%	12.2%	*<0.0001*
**Vit E (mg)**	1.9%	3.0%	2.0%	*0.2795*
**Ca (mg)**	0.6%	4.6%	46.9%	*<0.0001*
**Mg (mg)**	2.1%	8.0%	25.2%	*<0.0001*
**Zn (mg)**	57.2%	72.5%	90.2%	*<0.0001*
**Iron (mg)**	82.2%	91.9%	95.4%	*<0.0001*
**P (mg)**	18.3%	44.7%	88.5%	*<0.0001*
**K (mg)** [2]	0.1%	0.0%	0.2%	*0.3993*
	Cheese & yogurt subgroup			
	**Low** [3]	**Moderate** [3]	**High** [3]	***p-value***
	n = 452	*n = 1202*	*n = 447*	
**Vit A (RAE)**	42.9%	52.8%	64.7%	*<0.0001*
**Vit B6 (mg)**	73.9%	82.0%	86.6%	*<0.0001*
**Vit B12 (µg)**	74.8%	84.9%	92.8%	*<0.0001*
**Vit C (mg)**	70.4%	77.7%	81.0%	*0.0004*
**Vit D (µg)**	2.9%	2.4%	2.0%	*0.7016*
**Vit E (mg)**	3.5%	2.0%	2.2%	*0.1812*
**Ca (mg)**	4.7%	8.1%	26.6%	*<0.0001*
**Mg (mg)**	6.9%	8.3%	13.0%	*0.0027*
**Zn (mg)**	54.0%	68.8%	89.0%	*<0.0001*
**Iron (mg)**	79.9%	89.4%	95.8%	*<0.0001*
**P (mg)**	20.8%	40.1%	72.3%	*<0.0001*
**K (mg)** [2]	0.4%	0.0%	0.0%	*0.0259*

[1] Low, moderate, high intakes: <20%, 20%–<50%, ≥50% of the recommended 3 servings of total dairy/day derived from milk; [2] Percent meeting AI for potassium (K); [3] Low, moderate, high intakes: <10%, 10%–<30%, ≥30% of the recommended 3 servings of total dairy/day derived from cheese and yogurt.

Supplemental Table S4. Percent of 14–18 year-old girls meeting Estimated Average Requirements (EAR) for micronutrients according to meat subgroup intake.

	Red meat			
	**Low [1]**	**Moderate [1]**	**High [1]**	***p-value***
	n = 557	*n = 811*	*n = 733*	
**Vit A (RAE)**	56.7%	51.5%	52.4%	*0.1435*
**Vit B6 (mg)**	68.4%	80.3%	92.1%	*<0.0001*
**Vit B12 (µg)**	64.1%	86.2%	98.0%	*<0.0001*
**Vit C (mg)**	72.2%	75.0%	82.4%	*<0.0001*
**Vit D (µg)**	3.8%	2.0%	1.9%	*0.0557*
**Vit E (mg)**	1.3%	2.0%	3.7%	*0.0113*
**Ca (mg)**	13.1%	9.6%	11.7%	*0.1198*
**Mg (mg)**	9.2%	6.7%	11.5%	*0.0044*
**Zn (mg)**	41.3%	67.3%	94.5%	*<0.0001*
**Iron (mg)**	84.6%	86.1%	94.8%	*<0.0001*
**P (mg)**	32.0%	35.1%	59.5%	*<0.0001*
**K (mg) [2]**	0.2%	0.0%	0.1%	*0.5167*
	White meat (poultry and fish)			
	**Low [3]**	**Moderate [3]**	**High [3]**	***p-value***
	n = 1022	*n = 671*	*n = 408*	
**Vit A (RAE)**	53.5%	54.1%	51.0%	*0.5864*
**Vit B6 (mg)**	73.6%	86.7%	91.4%	*<0.0001*
**Vit B12 (µg)**	81.6%	86.9%	87.5%	*0.0022*
**Vit C (mg)**	73.2%	80.2%	80.4%	*0.0006*
**Vit D (µg)**	2.6%	2.4%	2.0%	*0.7487*
**Vit E (mg)**	1.7%	1.9%	4.9%	*0.0009*
**Ca (mg)**	12.9%	10.0%	9.3%	*0.0661*
**Mg (mg)**	8.0%	7.9%	13.2%	*0.0038*
**Zn (mg)**	64.2%	72.9%	79.4%	*<0.0001*
**Iron (mg)**	85.6%	91.7%	91.7%	*<0.0001*
**P (mg)**	39.8%	43.1%	49.8%	*0.0028*
**K (mg) [2]**	0.2%	0.0%	0.0%	*0.3476*
	Soy, nuts, legumes, eggs			
	**Low [4]**	**Moderate [4]**	**High [4]**	***p-value***
	n = 1144	*n = 687*	*n = 270*	
**Vit A (RAE)**	46.8%	60.3%	62.6%	*<0.0001*
**Vit B6 (mg)**	77.5%	85.4%	86.3%	*<0.0001*
**Vit B12 (µg)**	80.9%	89.4%	87.0%	*<0.0001*
**Vit C (mg)**	73.6%	81.1%	79.6%	*0.0006*
**Vit D (µg)**	2.0%	2.3%	4.4%	*0.0638*
**Vit E (mg)**	1.1%	1.8%	9.6%	*<0.0001*
**Ca (mg)**	9.4%	12.1%	17.4%	*0.0006*
**Mg (mg)**	4.2%	10.3%	25.9%	*<0.0001*
**Zn (mg)**	61.7%	78.6%	82.6%	*<0.0001*
**Iron (mg)**	84.5%	92.7%	96.3%	*<0.0001*
**P (mg)**	32.1%	52.8%	62.6%	*<0.0001*
**K (mg) [2]**	0.1%	0.0%	0.4%	*0.2452*

[1] Low, moderate, high: <25%, 25%–<50%, ≥50% of the recommended 5 one-ounce servings of total meat/day derived from red meat; [2] Percent meeting AI for potassium (K); [3] Low, moderate, high: <20%, 20%–<40%, ≥40% of the recommended 5 one-ounce servings of total meat/day derived from white meat; [4] Low, moderate, high: <10%, 10%–<20%, ≥20% of the recommended 5 one-ounce servings of total meat/day derived from soy, nuts, legumes, eggs.

Supplemental Table S5. Percent of 14–18 year-old girls meeting Estimated Average Requirements (EAR) for micronutrients according to grain subgroup intake.

	Whole grain intake			
	**Low [1]**	**Moderate [1]**	**High [1]**	***p-value***
	n = 999	*n = 532*	*n = 570*	
**Vit A (RAE)**	37.5%	59.6%	74.7%	*<0.0001*
**Vit B6 (mg)**	73.9%	84.6%	91.1%	*<0.0001*
**Vit B12 (µg)**	82.1%	86.7%	86.5%	*0.0180*
**Vit C (mg)**	70.1%	80.6%	85.1%	*<0.0001*
**Vit D (µg)**	0.8%	2.1%	5.6%	*<0.0001*
**Vit E (mg)**	1.9%	2.6%	3.0%	*0.3645*
**Ca (mg)**	6.4%	9.8%	21.2%	*<0.0001*
**Mg (mg)**	3.1%	7.0%	21.2%	*<0.0001*
**Zn (mg)**	63.7%	70.5%	80.4%	*<0.0001*
**Iron (mg)**	82.3%	93.1%	96.0%	*<0.0001*
**P (mg)**	32.0%	46.4%	58.3%	*<0.0001*
**K (mg) [2]**	0.1%	0.0%	0.2%	*0.6391*
	Refined grain intake			
	**Low [3]**	**Moderate [3]**	**High [3]**	***p-value***
	n = 559	*n = 667*	*n = 875*	
**Vit A (RAE)**	36.9%	52.0%	64.6%	*<0.0001*
**Vit B6 (mg)**	66.4%	81.1%	90.9%	*<0.0001*
**Vit B12 (µg)**	76.2%	83.2%	90.6%	*<0.0001*
**Vit C (mg)**	67.8%	77.7%	81.9%	*<0.0001*
**Vit D (µg)**	1.8%	1.4%	3.7%	*0.0074*
**Vit E (mg)**	1.6%	1.8%	3.3%	*0.0583*
**Ca (mg)**	3.9%	7.8%	18.6%	*<0.0001*
**Mg (mg)**	2.7%	5.3%	15.9%	*<0.0001*
**Zn (mg)**	49.2%	67.6%	84.9%	*<0.0001*
**Iron (mg)**	65.1%	93.9%	99.9%	*<0.0001*
**P (mg)**	16.3%	36.6%	64.5%	*<0.0001*
**K (mg) [2]**	0.2%	0.0%	0.1%	*0.5825*

[1] Low, moderate, high intakes: <5%, 5%–<10%, ≥10% of the recommended 6 servings of total grains/day derived from whole grains; [2] Percent meeting AI for potassium (K); [3] Low, moderate, high intakes: <75%, 75%–<100%, ≥100% of the recommended 6 servings of total grains/day derived from refined grains.

Supplemental Figure S1. Percent of 14–18 year-old girls meeting EAR according to combined effects of dairy and whole grains. Low (*vs.* high) dairy: <75% (*vs.* ≥75%) of recommended 3 dairy servings/day. Low (*vs.* high) whole grain intake: <10% (*vs.* ≥10%) of recommended 6 servings of total grains/day were derived from whole grains.

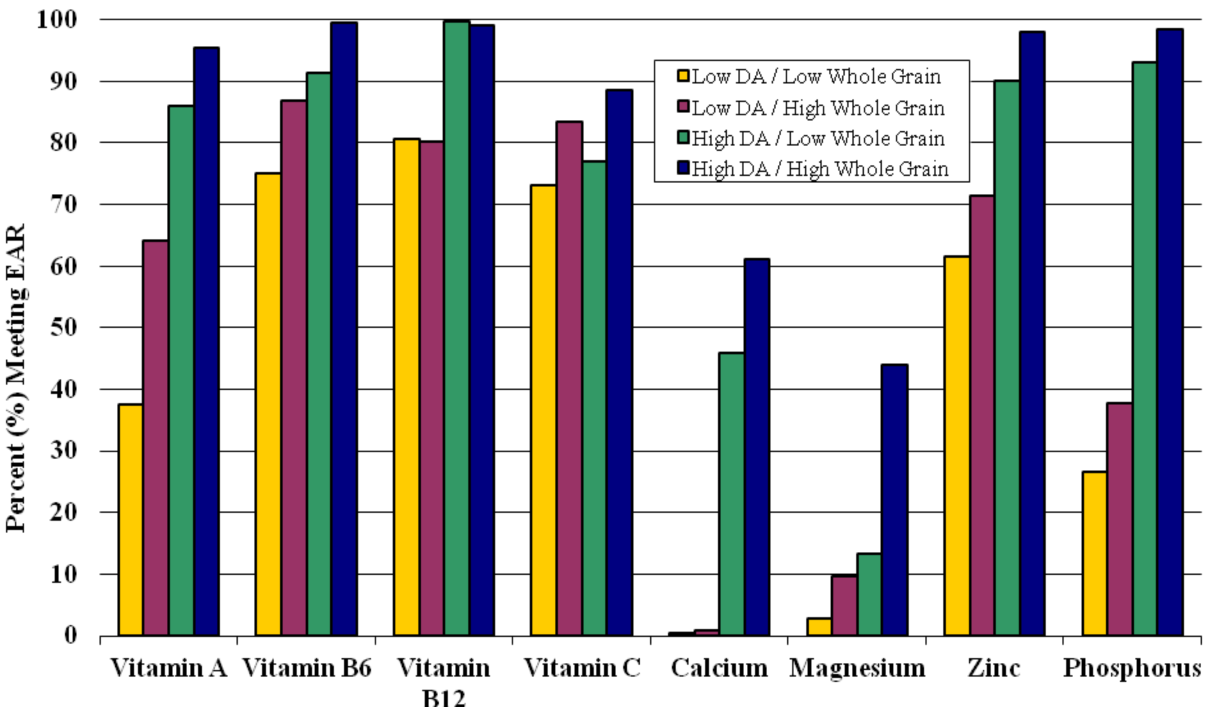

Supplemental Figure S2. Percent of 14–18 year-old girls meeting EAR according to combined effects of red meat and whole grains. Low (*vs.* high) red meat: <50% (*vs.* ≥50%) of recommended 5 one-ounce servings of total meat/day. Low (*vs.* high) whole grain intake: <10% (*vs.* ≥10%) of recommended 6 servings of total grains/day were derived from whole grains.

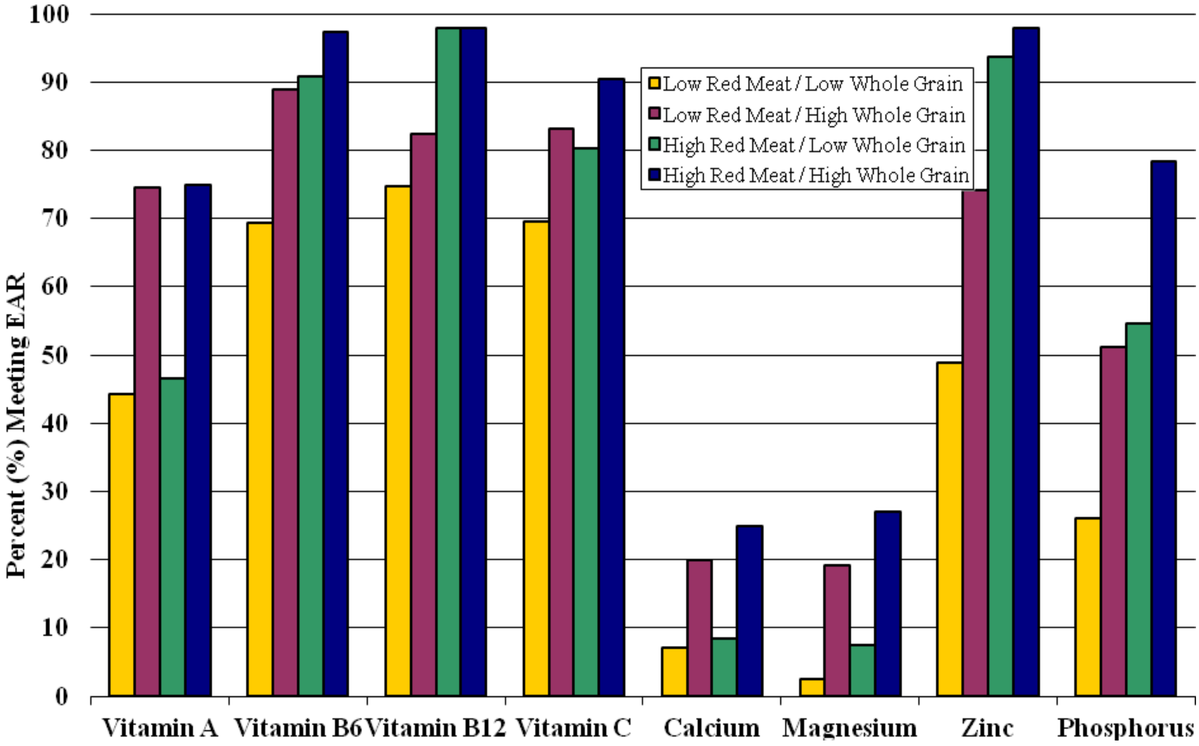

Supplemental Figure S3. Percent of 14–18 year-old girls meeting EAR according to combined effects of white meat (poultry and fish) and whole grains. Low (*vs.* high) white meat: <40% (*vs.* ≥40%) of recommended 5 one-ounce servings of total meat/day. Low (*vs.* high) whole grain intake: <10% (*vs.* ≥10%) of recommended 6 servings of total grains/day were derived from whole grains.

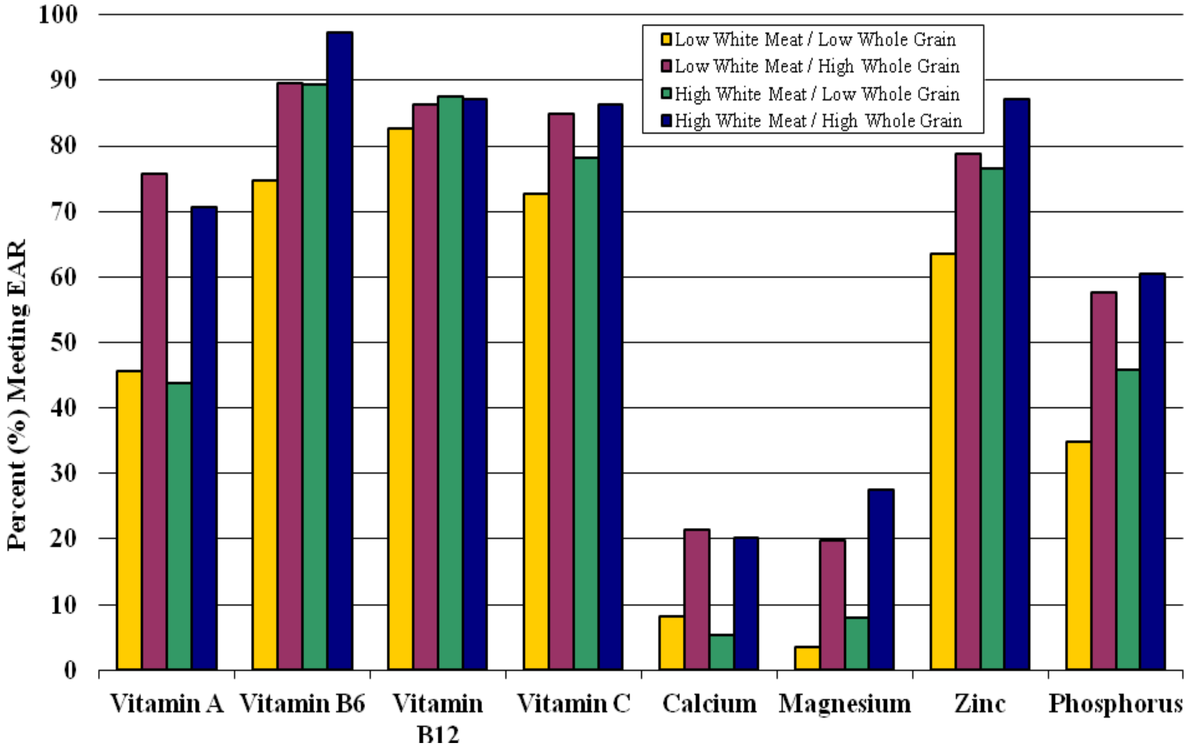

Supplemental Figure S4. Percent of 14–18 year-old girls meeting EAR according to combined effects of non-meat protein (eggs, soy, nuts, and legumes) and whole grains. Low (*vs.* high) non-meat protein: <10% (*vs.* ≥10%) of recommended 5 one-ounce servings of total meat/day. Low (*vs.* high) whole grain intake: <10% (*vs.* ≥10%) of recommended 6 servings of total grains/day were derived from whole grains.

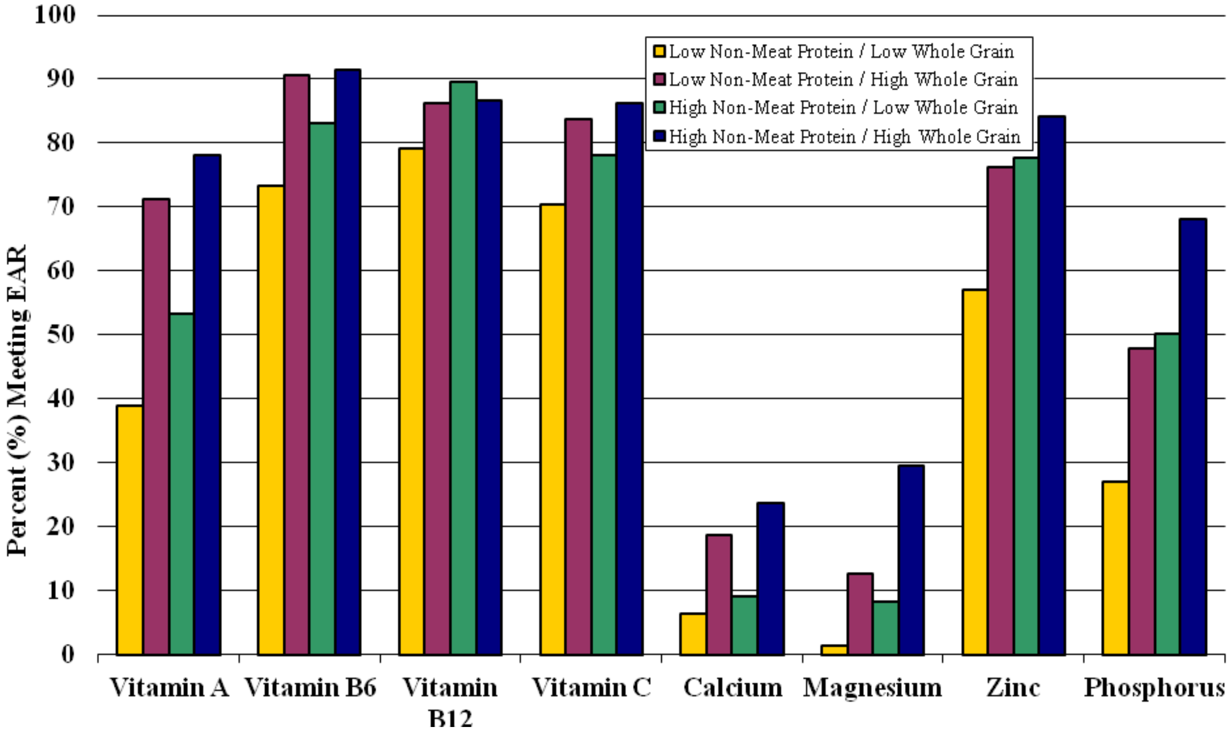

Supplemental Figure S5. Percent of 14–18 year-old girls meeting EAR according to combined effects of non-meat protein (eggs, soy, nuts, and legumes) and fruits and vegetables. Low (*vs.* high) non-meat protein: <10% (*vs.* ≥10%) of recommended 5 one-ounce servings of total meat/day. Low (*vs.* high) fruit and vegetable intake: <50% (*vs.* ≥50%) of recommended 8 servings of total fruit and vegetables/day.

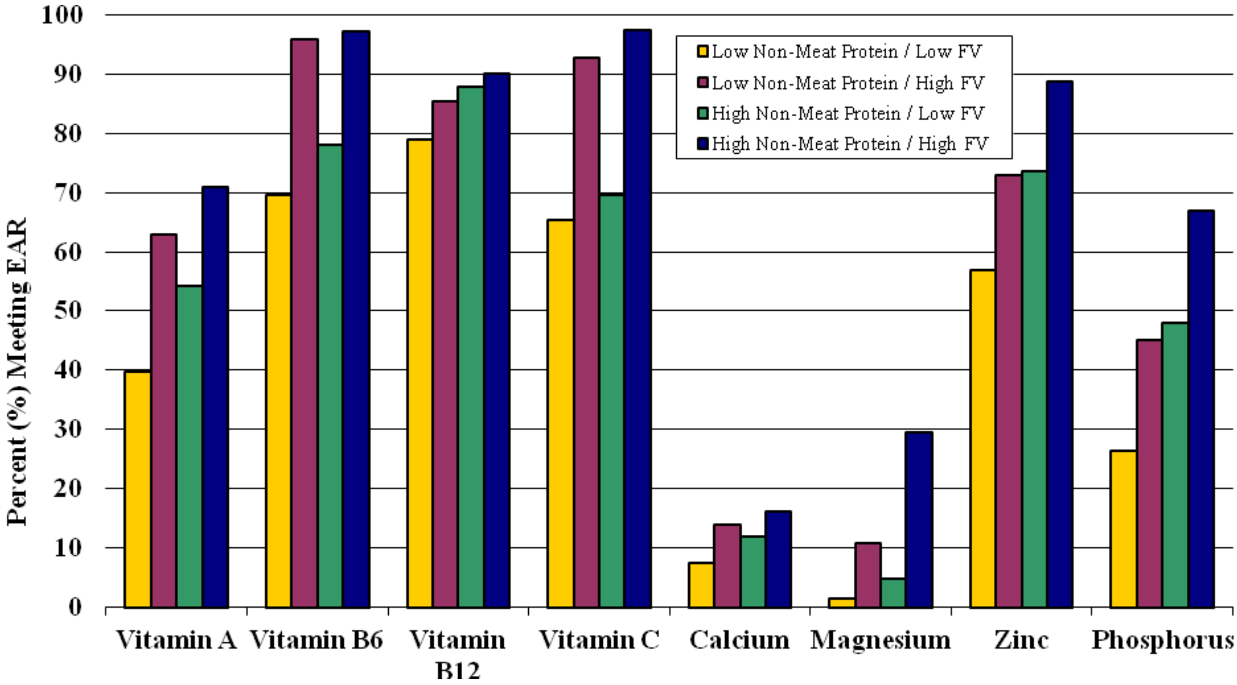

Supplement: Supplementary File 1: — Supplementary File (PDF, 294 KB) [file nutrients-04-01692-s001.pdf]
